# Radiolocation Devices for Detection and Tracking Small High-Speed Ballistic Objects—Features, Applications, and Methods of Tests

**DOI:** 10.3390/s19245362

**Published:** 2019-12-05

**Authors:** Marek Brzozowski, Mariusz Pakowski, Mirosław Nowakowski, Mirosław Myszka, Mirosław Michalczewski

**Affiliations:** Laboratory Testing of Radar and Aviation Technology, Air Force Institute of Technology (AFIT), 01-494 Warsaw, Poland; miroslaw.nowakowski@itwl.pl (M.N.); miroslaw.myszka@itwl.pl (M.M.); miroslaw.michalczewski@itwl.pl (M.M.)

**Keywords:** radar, research radar, high-speed ballistic objects tracking, radar field tests, GPS applications

## Abstract

This article describes radiolocation devices dedicated to the detection and tracking of small high-speed ballistic objects and multifunctional radars. This functionality is implemented by applying space search technology and adaptive algorithms for detection and tracking of air objects in parallel with classic search and tracking of objects in controlled airspace. This article presents examples of the construction of both types of devices produced by foreign companies and Polish industry. The following sections present methods for testing radars with the function of tracking small high-speed ballistic objects along with examples of results of observations of combat ammunition.

## 1. Introduction

We presented the issue of radars capable of detecting and tracking high-speed ballistic objects, as well as issues related to the specifics of research and testing of such devices at the conference Metro Aerospace 2019, in Turin. The presented issues were met with great interest from the conference participants and became a source of information for many people about radar devices manufactured by the Polish industry.

This article expands the subject of the publication "Radars with the function of detecting and tracking artillery shells—selected methods of field testing", published in Metro AeroSpace 2018 proceedings [1].

Despite continuous technological development, the role of classic artillery in a contemporary battlefield is not diminishing. If we observe this in relation to the conflicts conducted in recent years, it is easy to notice that most activities of infantry units take place under covering fire from lighter or heavier artillery. Both cases, which aim to destroy a detected enemy and protect our positions, still require using sufficient fire power to quickly and effectively incapacitate the enemy. The key to effective use of artillery is to quickly and precisely determine the coordinates of the position of targets and to verify the accuracy and effectiveness of the conducted firing [2].

In this area, modern technology offers the following two most popular solutions: Unmanned aerial vehicles (UAV) equipped with optoelectronic observation sensors and specialized radiolocation devices intended for detecting, tracking, and calculating the trajectory parameters of high-speed ballistic objects [3]. Both methods have their advantages and disadvantages. This paper only features the characteristics of radars. The discussion features methods of detecting high-speed ballistic objects using radiolocation sensors and examples of existing technological solutions used globally and in the Republic of Poland. This paper also contains a detailed discussion of the methods of testing specialized ballistic radars or multifunctional radars with the ability to detect and track high-speed ballistic objects viewed as one of their operating modes. It also features examples of the results of field testing conducted using real high-speed ballistic objects [1,4].

We present research methods and example results related to Polish radars developed for the needs of the Polish Armed Forces. From a scientific point of view, it would be desirable to present many technical parameters that characterize individual sensors and compare the effects of different sensors depending on the technical solutions used in them, but this is impossible because the detailed technical parameters and test results of these devices are secret.

## 2. Materials and Methods

The latter part of this paper, aside from a short characterization of radiolocation devices intended for detecting and tracking high-speed ballistic objects, includes a presentation of testing methods used at the Air Force Institute of Technology (AFIT) for the purpose of field testing to check the most important parameters of such devices. The most frequently used verifications are as follows: minimum and maximum distances, minimum and maximum height, maximum elevation angle of detecting for a given type of detected object, and the accuracies of determining the points of origin (POO) and points of impact (POI) [2]. 

### 2.1. Detection and Tracking of High-Speed Ballistic Objects Using Radiolocation Sensors

The issue of detecting and tracking high-speed ballistic objects has been a significant challenge for radiolocation. Because these objects achieve high velocities and move at relatively small heights above the horizon, the device that ensures their effective detection and tracking must quickly scan the horizon (several times per second) in order to immediately detect the flight object and track its flight path, i.e., observe space in a broad range of elevation angles with the radar beam [3]. 

These capabilities have appeared along with the development of antennas with an electronically controlled pencil beam. Such antennas allow for very fast beam movement both in the azimuth and elevation planes. This searches the entire observation sector with an information refreshing time of less than 1 s. The algorithms for searching ballistic objects are usually optimized to search a narrow elevation sector right above the horizon. This allows for the optimal use of the radar’s time budget. Application of this method is possible because after detecting the echo, the radar automatically switches to the detection mode using additional lightings with a pencil beam, which obtains information about the high-speed ballistic object’s flight trajectory, optimized in terms of the ballistic calculations [5,6]. On the basis of subsequent object detection, the ballistic calculator determines the appropriate ballistic model to calculate its full flight trajectory. The calculated trajectory is then applied to the terrain’s digital model, which determines the POO and POI’s coordinates. Antennas with an electronically controlled beam are made using various technologies, starting with passive antennas with phase-frequency control and ending with active antennas made in the form of transmit and receive module array with full digital control of the transmission and reception characteristics [7]. The only disadvantage of both types of older and modern antennas is that the electronic characteristic control is realized only in a limited sector of azimuth and elevation angles. The limitation of the azimuth sector, usually to approximately ±45° in relation to the antenna’s normal aperture, is especially troublesome. This type of antenna is, therefore, usually rotated mechanically in the azimuth plane and such radar’s operational use imposes the need to determine the sector of responsibility earlier. The POO and POI coordinates designated by the radar are usually transmitted to an automated command and control system.

**Figure 1:**
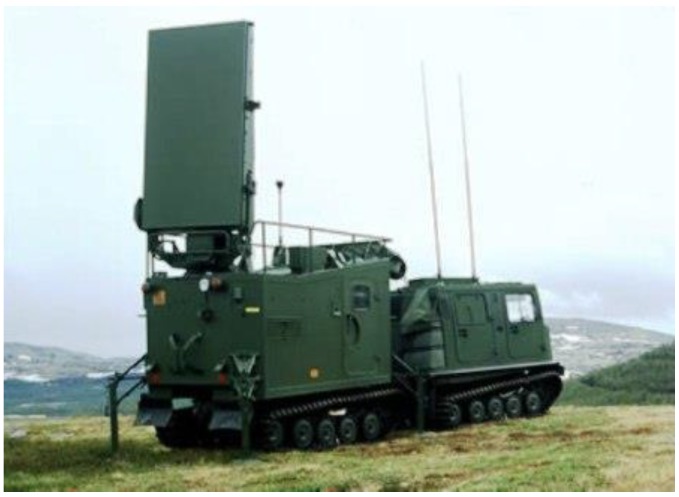
ARTillery HUNting Radar (www.radartutorial.eu) [8].

One of the most popular examples of a radar intended for tracking high-speed ballistic objects and detecting launchers positions is ARTHUR (ARTillery HUnting Radar, Ericsson Microwave Sytsem AB, Mölndal, Sweden) (Figure 1) developed and manufactured by Ericsson Microwave Systems (currently SAAB Microwave Systems) [2].

The radar is a device characterized by high mobility, able to quickly and effectively move in a combat operations area. It operates in the C band (4 to 8 GHz) and possesses a flat passive antenna with electronic beam scanning in both planes. In elevation, the antenna beam is controlled by changing the probing signal’s frequency, whereas in the azimuth the control takes place using phase shifters [8].

The width of the azimuth sector in which the electronic beam scanning is conducted amounts to 90°. The radar’s antenna setting towards the selected direction is executed by its mechanical rotation. The radar detections are analyzed by an advanced software that determines the POO and POI coordinates with consideration of the terrain’s digital model and computation of commands for the co-operating military units. In order to execute the aforementioned functions, the radar must precisely determine its position in the terrain. This is done with the use of an advanced navigation system consisting of an inertial module and a GPS receiver [2].

Other examples of radars intended for detecting high-speed ballistic objects and launcher positions include: AN/TPQ-37, AN/TPQ-47, and AN/TPQ-50 developed by Thales Raytheon Systems, COBRA (COunter Battery Radar) [2] developed and manufactured in co-operation with French, German, and Turkish companies, as well as the Chinese SLC-2 Fire Finding Radar structure [2].

### 2.2. Polish Radars with the Function of Detecting and Tracking Small High-Speed Ballistic Objects

**Figure 2:**
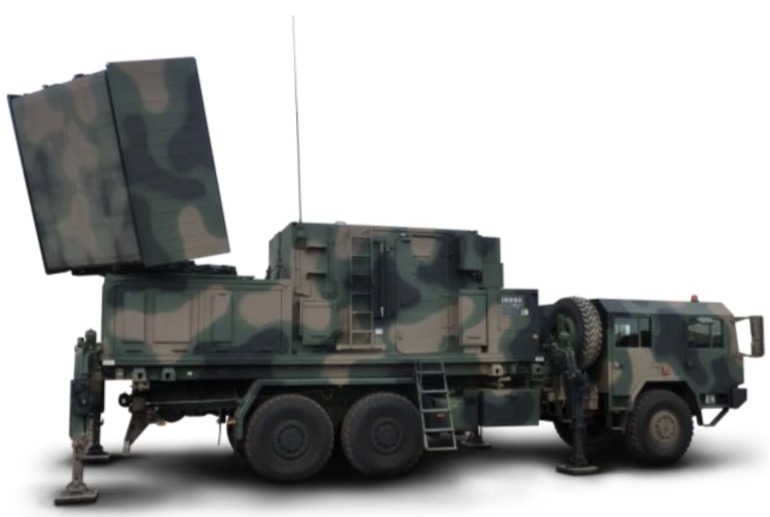
Radiolocation Artillery Reconnaissance Unit LIWIEC (www.pitradwar.com).

Radars produced by Polish companies also detect and track small high-speed ballistic objects and determine the POO and POI. An example of a device especially dedicated for such tasks is the Radiolocation Artillery Reconnaissance Unit LIWIEC [9] (Figure 2). It is embedded on a wheeled chassis and is equipped with a passive flat antenna with a two-plane electronically controlled beam. The maximum width of the observed azimuth sector amounts to 90°. The frequency of scanning and refreshing information about each element in the sector amounts to 0.5 s. The antenna’s mechanical rotation in the azimuth allows for sector control in a 270° width. The maximum observation angle of objects in elevation amounts to 20° [9].

The LIWIEC radar can be used to protect important objects and support artillery operation in the following scope:

- automatic detection and tracking of high-speed ballistic objects;
- determination of the coordinates of launcher positions (single and grouped);
- automatic classification of flight object type and launcher position and type;
- determination of the POI coordinates;
- transmission of information to automated command and control systems.

The main advantage of this radar is that its operator can adapt space scanning algorithms to current needs, and, in addition, the automatic tracking system of detected objects can force more frequent scanning of new objects, which greatly accelerates the calculation of the ballistic trajectory of the tracked object. On the basis of the calculated trajectory, the radar identifies the type of detected object (including its launcher type) and accurately determines the POO and POI before the detected object reaches its target.

Aside from small high-speed ballistic objects, the LIWIEC radar detects and tracks aircrafts, helicopters, unmanned aerial vehicles, and land-based mechanical vehicles [9].

A different perspective on the problem of detecting small high-speed ballistic objects and especially mortars is applied in the SOŁA and BYSTRA radars. The radars are intended for air defense forces to control airspace in the area of land troops operations. Both devices search the airspace by mechanically rotating the antenna in the azimuth plane. The application of relatively high antenna rotation speeds (30 and 60 rpm) guarantee the ability to detect and track small high-speed ballistic objects with sufficient accuracy for the ballistic calculation algorithm to estimate the complete object trajectory based on the sample of over a dozen detections, and therefore determines the POO and POI with satisfactory accuracies. Especially good results of such a method for detection and tracking are achieved in relation to mortar [4].

**Figure 3:**
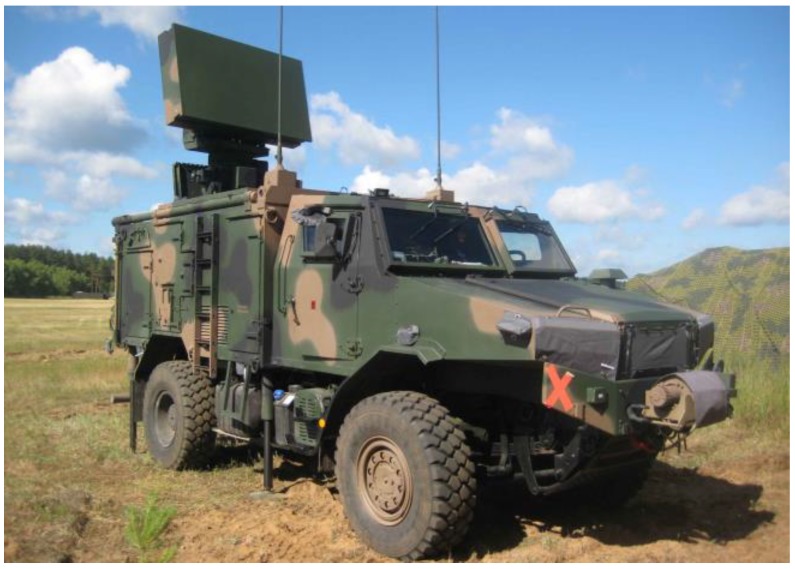
Redeployment-capable radiolocation station SOŁA (www.pitradwar.com).

The SOŁA redeployment-capable radiolocation station (Figure 3) is a short-range radar operating in the S band, intended for the SHORAD (short range air defense) systems. It ensures the detection of aerial objects, including unmanned air vehicles, helicopters, and small high-speed ballistic objects. It is a three-dimensional radar with an antenna rotated mechanically along the azimuth and a beam controlled electronically in the elevation. Depending on the operating mode, the radar’s antenna can be rotated with a speed of 30 or 60 rpm. The radar is embedded in an armored vehicle with very good off-road performance. The data on the detected objects is transmitted from the radar to the automated command system via digital radio link [9].

**Figure 4:**
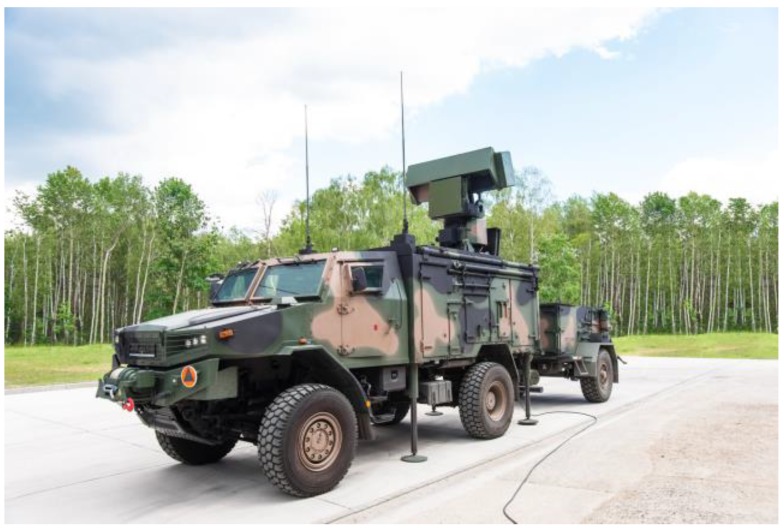
Redeployment-capable radiolocation station BYSTRA (www.pitradwar.com) [9].

The BYSTRA (Figure 4) is a multifunctional and multitask radar with versatile capabilities and applications, possessing the ability to detect and track typical aerial threats, such as aircrafts and helicopters (also hovering), unmanned air vehicles, and small high-speed ballistic objects, particularly mortars. The radar uses state-of-the-art technological solutions, including: active antenna with semiconducting transmitting modules and electronically controlled transmission beam position, digital receiving beams formation, digital synthesis, signal encoding and matched filtering, coordinate estimation supported by the algorithm limiting the multipath effects, and finally tracking system using the multiple hypothesis algorithm. The applied solutions achieving quick radiolocation information refreshing of no more than 2 s [9].

Electronic control of the position of the antenna beam in the azimuth plane allows the system to automatically track air objects and generate re-scanning requests for newly detected objects in the same antenna rotation. This is a very important property of radar, which very quickly eliminates false ballistic trajectories based on the detection of passive or active interference.

The SOŁA and BYSTRA radars are perfectly matched for covering and protecting important objects and areas because, during normal operation of controlling the airspace around the object, they additionally ensure the execution of the helicopter detection function, including hovering helicopters, as well as the detection and tracking of small high-speed ballistic objects including the POO and POI coordinates’ estimation. This functionality provides the personnel of the protected object early warning about the threat detected. 

### 2.3. Small High-Speed Ballistic Objects Detection Zone Verifications

The design and production of radars capable of detecting and tracking small high-speed ballistic objects and calculating their ballistic trajectory parameters requires developing specific research methods to objectively evaluate their technical parameters. Generally, such radars require conducting actual observations of shelling from many types of launchers. The planning of such tests requires simultaneous consideration of many various factors, including the following:

- expected values of the tested radar parameter;
- firing capabilities of the given launcher;
- available military trying area;
- safety zones military trying area;
- possibility of finding a proper place of radar operation in relation to launcher position and firing targets.

In the case of conducting testing to verify a small high-speed ballistic object detection zone, mortar shelling is most often used. This means of launcher fires ballistic objects to both small and large heights, in a broad range of distances and does not require too large of a safety zone. The most difficult element in preparing the tests for the conditions of a specific trying area is most often finding a suitable place of operation for the tested radar. Usually, the area of firing means that location of launchers and field of fire on a trying area are imposed by the adopted safety zones, whereas the terrain outside of the tactical strips is covered by forest. For this reason, the location of equipment in the field can only approximately correspond to the theoretical assumptions. Prior to the small high-speed ballistic object detection, each radar undergoes testing with the use of unmanned or classical aircrafts equipped with GPS receivers that record their flight trajectory. The comparison of radar detections with the recorded flight trajectory determines the accuracy of estimation of the detected object’s coordinates (azimuth, distance, elevation angle, and height of flight). The sequence of testing analyzes the small high-speed ballistic object detection with consideration of the earlier designated estimation errors.

#### 2.3.1. Verification of the Minimum Distance and Minimum Height of Small High-Speed Ballistic Objects Detection and Tracking

**Figure 5:**
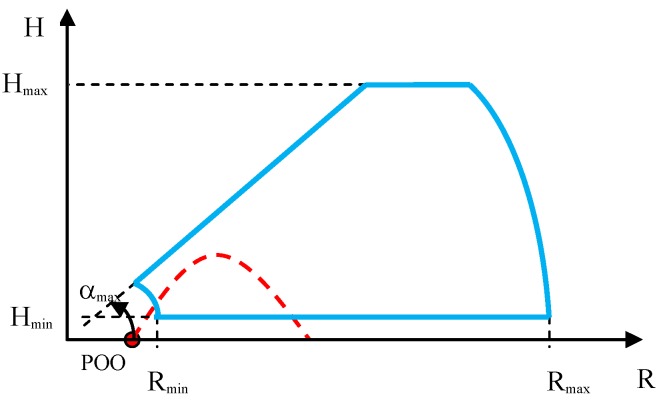
Sketch of the method of observing the mortar firings for the purpose of verifying the minimum detection distance and height. Start of the coordinates system in the radar position. Points of origin (POO), mortar position; radar detection characteristic, blue color; and desired ballistic objects flight trajectory, red color.

In the case of verification of the minimum distance and minimum height of small high-speed ballistic objects detection and tracking, the mortar’s position must be located in the radar’s dead zone. The ballistic objects must be fired into the radar’s characteristic, at a small angle, so that the radar is capable of observing them continuously along the entire flight trajectory. Such a test requires realization of several dozen firings in order to evaluate the minimum ballistic objects detection distance and height in a statistical manner. The test sketch is presented below (Figure 5).

**Figure 6:**
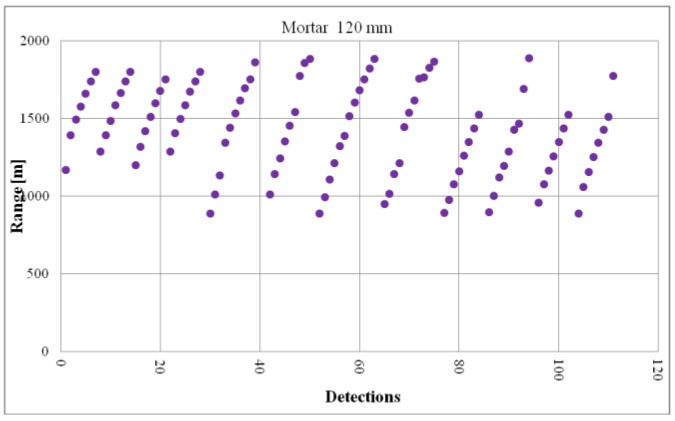
Example of mortars detections recorded during the verification of the minimum detection distances.

Figure 6 presents a set of data recorded during the testing of the minimum ballistic object detection distance. The graph presents the object detection distances from subsequent firings. Then, the minimum distance values were selected from the entire observation and this set of data was used to estimate the average value and standard deviation, as well as the bottom and top confidence interval limit. The calculated parameters allowed for an objective evaluation of the actual value of the minimum detection distance of the tested device.

**Figure 7:**
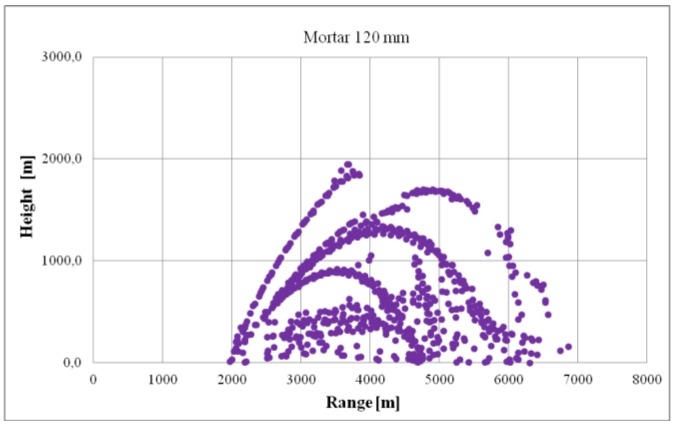
Example of mortar detections recorded during the verification of the parameter minimum detection heights.

Figure 7 presents a set of data recorded during the testing of the minimum ballistic object detection height. The graph presents detections in the following coordinates: distance and height. Due to the verification’s specificity, the radar’s position in relation to the mortar’s position and target of fire was especially important. The radar must be able to observe the ballistics’ entire flight trajectory without any terrain obstacles. The detections of both ascending and descending flight objects were used for the evaluation of the parameter minimum detection height. From the complete set of detections, detections with minimum height values were selected. Such a dataset was used to conduct statistical calculations analogously to the minimum detection distance parameter.

#### 2.3.2. Verification of the Maximum Elevation Angle for the Detection and Tracking of Small High-Speed Ballistic Objects

**Figure 8:**
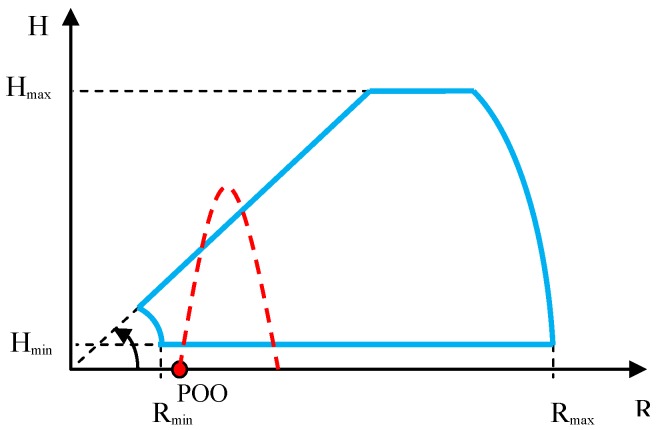
Sketch of the method of observing the mortars for the purpose of verifying the maximum angle of detection in elevation. Start of the coordinates system in the radar position. POO, mortar position; radar detection characteristic, blue color; desired flight trajectory, red color.

The sketch of firings conducted for the purpose of this test is presented in Figure 8. In order to obtain correct results from the observation of firings, it is necessary to carefully select the distance between the radar and the mortar’s firing position, with consideration of the expected maximum radar detection angle in elevation and distance, as well as the maximum flight height of the fired ballistic objects.

**Figure 9:**
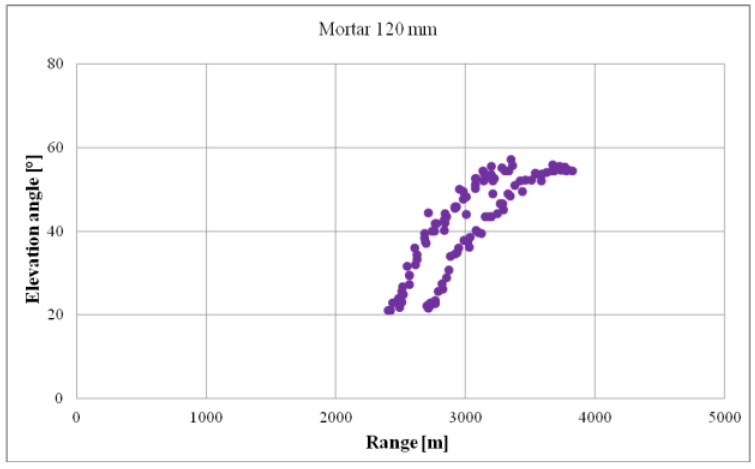
Example of mortar detections recorded during the verification of the maximum angle of detection in elevation.

Figure 9 presents the results of firings conducted for the purpose of verifying the maximum elevation angle. Then, detections with maximum elevation angle values were selected from the entire observation and statistical calculations were conducted with reference to the obtained dataset by designating the estimation of the sought parameter.

#### 2.3.3. Verification of the Maximum Height and Distance of Detection and Tracking of Small High-Speed Ballistic Objects 

**Figure 10:**
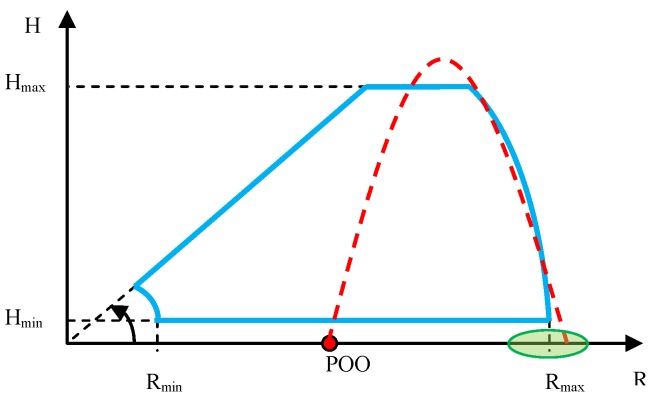
Sketch of the method of observing the mortars for the purpose of verifying the maximum detection height and distance. Start of the coordinates system in the radar position. POO, mortar position; radar detection characteristic, blue color; desired ballistics objects flight trajectory, red color; impact zone, green color.

The sketch of verification of the maximum height and distance of detection and tracking of mortars is presented in Figure 10.

The firings conducted for the purpose of verifying the maximum detection height must feature a large angle of mortar barrel elevation, with simultaneous use of the maximum propelling charge. This type of firing does not ensure sufficient range. In practical testing, it is, therefore, necessary to conduct separate firings at the maximum flight altitude and at the maximum detection range. The last type of test requires setting the mortar’s barrel at an angle near 45°.

**Figure 11:**
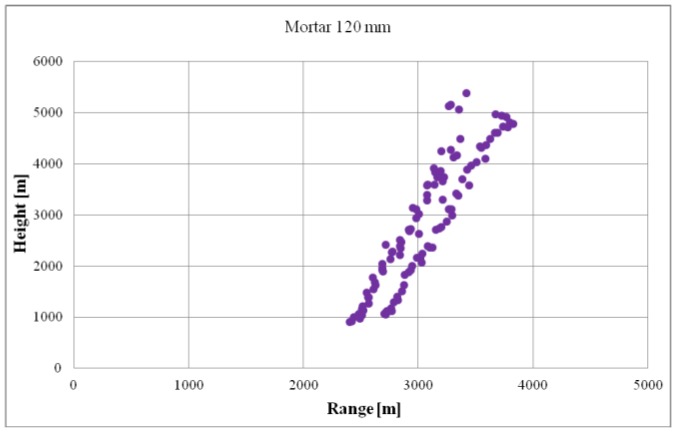
Example of mortar detections recorded during the verification of the parameter maximum detection height.

Figure 11 presents detections of ballistics objects fired in a manner allowing them to reach the highest flight altitude. The lack of descending trajectory was caused by the applied operating mode of the tested radar in which the search and initialization of flight objects tracking were only conducted in a small value of elevation angles.

**Figure 12:**
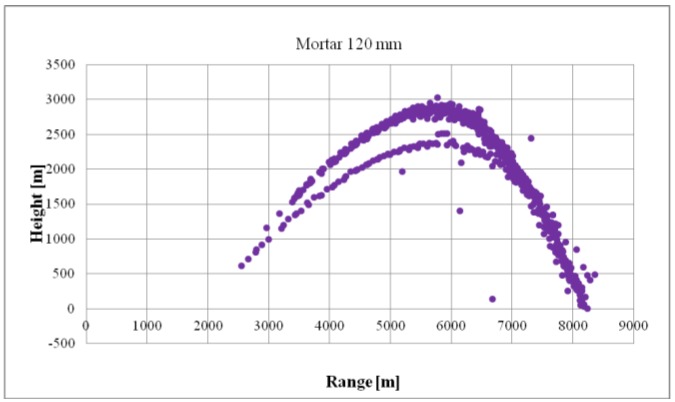
Example of results of verifying the maximum detection distance of mortars, i.e., height in the distance function.

Figure 12 presents detections of ballistics objects fired with launcher settings that ensured the maximum flight range. As shown in the figure, firing in such a manner did not cause the ballistic objects to exit the radar’s observation zone.

### 2.4. Verifications of the Accuracy of Determining the Coordinates of POO and POI

**Figure 13:**
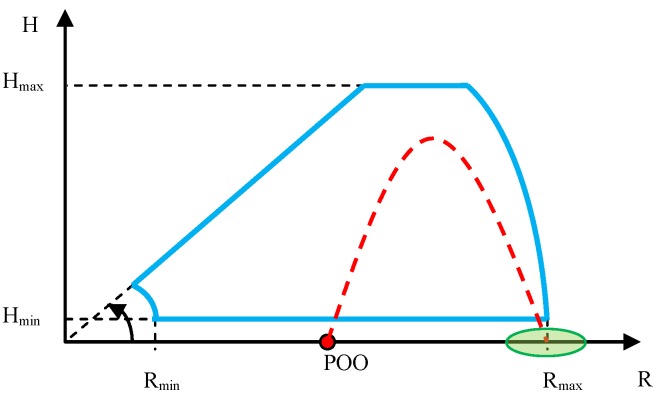
Sketch of the method of observing ballistic objects moving away from the radar for the purpose of verifying the accuracy of the POO and points of impact (POI). Start of the coordinates system in the radar position. POO, launcher position; radar detection zone, blue color; desired flight trajectory, red color; impact zone (POI), green color.

Verification of the accuracy of determining the coordinates of launchers positions (POO) and points of impact (POI) of ballistic objects when the radar observes objects that are moving away. This method controls the execution of the firing task at much greater distances than by optical observation instrumentation. A radar deployed on an observation position far from the enemy is also more difficult to destroy than an observation UAV (unmanned air vehicle), which in order to evaluate the firing effectiveness must fly near the area of the shelled target, and thus can be easily destroyed by the enemy. The sketch of the method of executing the test is presented in Figure 13.

In order to evaluate the radar’s POO estimation accuracy, the launcher position’s coordinates are measured with the use of a surveying GPS receiver from Spectra Precision, type Epoch 50 (Figure 21). Measurements are conducted in the differential mode with the use of data derived from the ASG-EUPOS network’s reference station. Such a measurement allows for achieving positioning accuracy of no less than 20 to 30 cm, and thereby assumes the measured coordinates as true coordinates. The coordinates obtained this way are then compared with the results of the POO estimation calculated by the tested radar. The estimation accuracy analysis is conducted in the UTM (Universal Transverse Mercator) rectangular coordinates system.

**Figure 14:**
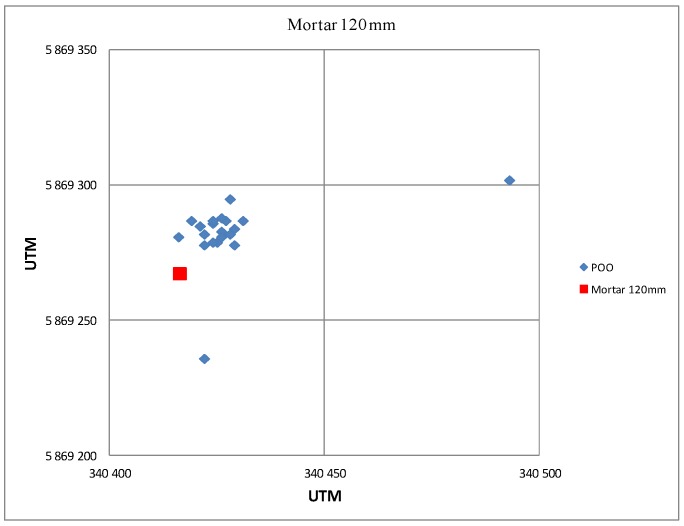
Example of estimations of the POO of 120 mm mortars. Firing in the direction from the radar.

Figure 14 presents the results of POO estimation conducted based on the observation of a 120 mm mortar firing. The red color is used to mark the mortar positions, whereas the blue markers are the radar’s POO coordinates estimation conducted based on the observation of subsequently fired ballistic objects.

**Figure 15:**
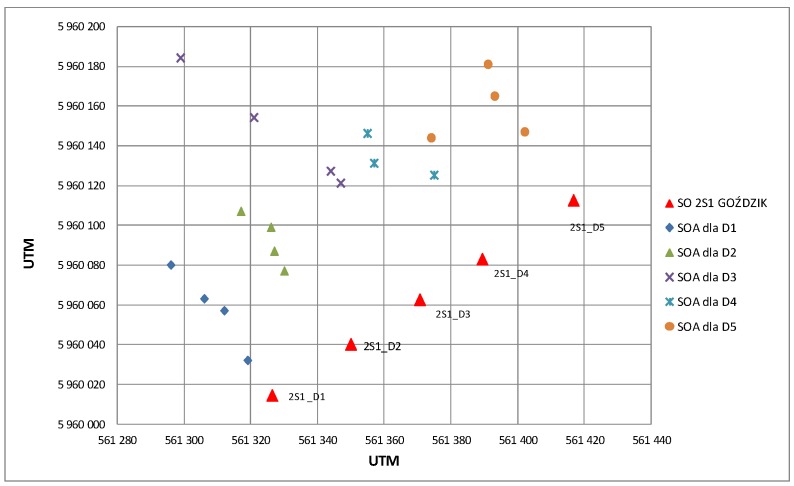
Example of estimations of the POO of 122 mm caliber cannon. Firing in the direction from the radar.

Figure 15 presents results of the radar’s observations of firings of a 122 mm caliber cannon subdivision. In the figure, red markers represent the positions of particular cannons, measured by the GPS receiver. Other markers illustrate subsequent POO estimations and are assigned with colors to particular cannons. In principle, the flight trajectories of ballistic objects fired from the cannon are flatter than mortar flight trajectories. This impairs the conditions of radar observation of the flying objects and the conditions of estimating the ballistic trajectory, which due to its flattening, makes it more difficult to precisely designate the intersections with the digital terrain map. Consequently, the estimated POO demonstrates a relatively large spread in the axis compliant with the firing direction.

The analysis of the POI coordinates estimation accuracy of the tested radar is conducted similarly to the POO accuracy analysis. However, it features an essential difficulty in the form of the necessity to find the particular impact points after the firing and to measure their positional coordinates using the GPS receiver. The measurement itself is conducted similarly as in the case of measuring the firing mean position with the use of the surveying differential Epoch 50 receiver.

In order to find and identify the point of impact of each of the fired object, the firings must be executed in a special manner, i.e., subsequent objects must be fired not at the same target, but with a slight displacement, for example, 20 to 30 m left or right. Additionally, it is necessary to note the results of localizations conducted by military observers with the use of laser rangefinders. Such tests are always conducted by the polygon services in order to control compliance with safety conditions. The practice of field testing also shows that if the tested radar allows for stable tracking of the ballistic objects, it is necessary to note the estimated coordinates of particular POIs and use them to find particular points of impact. Such data can be entered into the manual GPS receiver and used in combination with the GO TO function to find the firing area and particular impact points in a relatively short time. During the searching of impact points, it is necessary to take special care, comply with the safety principles in force on the training area, and not take any actions without making arrangements with persons responsible for safety during the executed firings. 

**Figure 16:**
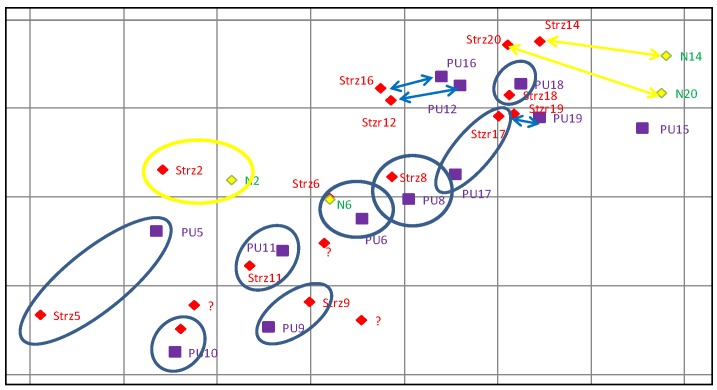
Illustration of the POIs of 120 mm mortars (purple color) estimated by the radar, GPS coordinates of the found points of impact (red color), and explosion localization by the military observers (yellow color).

Figure 16 presents the illustration of the position of POIs estimated by the radar, impact points determined by observers, actual points of impact found in the area and measured with the GPS receiver, as well as the matching markers of particular POI estimations for GPS measurement purposes. The matching of particular POIs with actual points of impact requires a detailed analysis of all datasets, because the actual points of impact can differ substantially from the adopted targeting scheme and the occurring POI estimation errors can in some cases suggest a better matching of the impact points of another object than the object actually observed.

**Figure 17:**
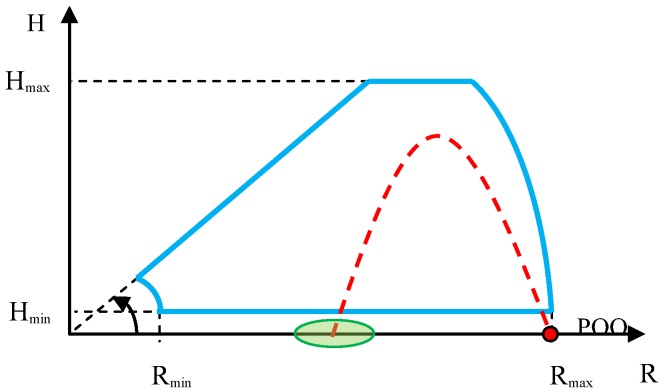
Sketch of the method of observing ballistic objects moving towards the radar for the purpose of verifying the accuracy of the POO and POI localization. Start of the coordinates system in the radar position. POO, mortar position; radar detection characteristic, blue color; desired flight trajectory, red color; impact zone (POI), green color.

Verification of the accuracy of determining the coordinates of the launcher position (POO) and the points of impact (POI) of ballistic objects when the radar observes objects that are moving towards it. The scheme of such verifications is presented in Figure 17.

The observation of firings executed in the direction of the radar requires particularly careful planning. On one hand, the radar must be placed in a suitable position, free of any terrain obstacles in the observation sector, at a distance ensuring the ability to detect flying objects. On the other hand, the test safety conditions must be respected, which means that the radar must be located at a suitable distance from the impact zone and the point of maximum theoretical range of the tested launcher. The reconciliation of these conditions is sometimes difficult and requires strict cooperation of the testing team with the trying area services.

**Figure 18:**
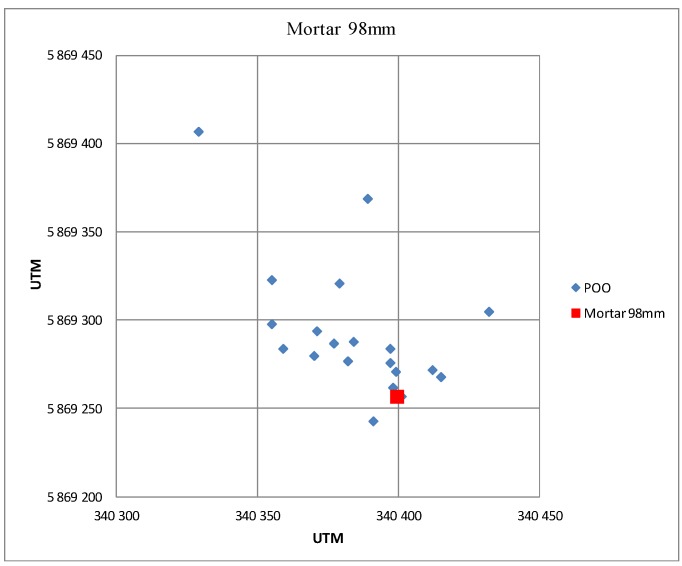
Example of estimations of the POO of 98 mm caliber mortar. Firing in the direction of the radar.

Figure 18 presents the results of POO estimation of a 98 mm mortar, obtained during the observation of objects flying in the direction of the radar.

**Figure 19:**
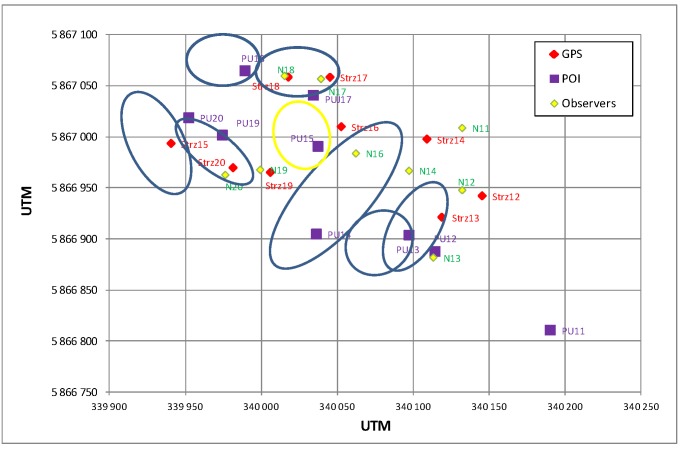
Illustration of the POIs of 98 mm mortars (purple color) estimated by the radar, GPS coordinates of the found points of impact (red color), and explosion localization by the observers (yellow color).

Figure 19 presents the results of POI estimation of a 98 mm mortar, obtained during the observation of ballistic object fired to the direction of the radar.

**Figure 20:**
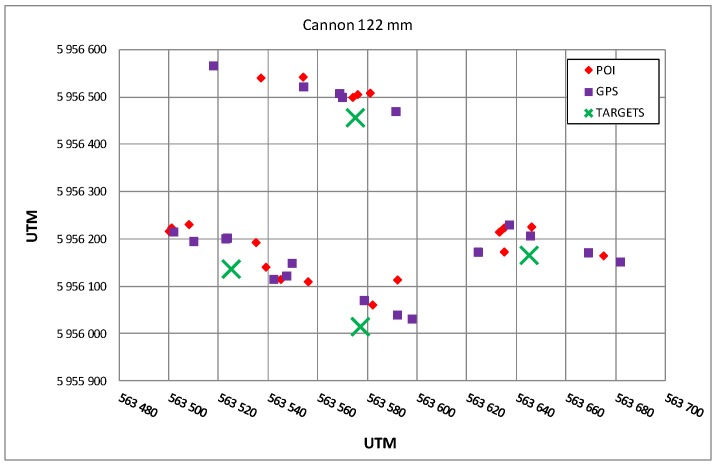
Illustration of the POIs of 122 mm caliber cannon (purple color) estimated by the radar, GPS coordinates of the found points of impact (red color), and coordinates of the targets (green color).

Figure 20 presents the comparison of the results of estimation of POI coordinates obtained during the observations of firings of a 122 mm caliber cannon. The firing was executed at four different targets that are also marked in the figure.

The presented results of field testing are illustrative, and their aim is only to illustrate the specifics of particular tests. The detailed results of verifications of the tactical and technical parameters of particular devices are confidential and will not be published.

### 2.5. Special Equipment

The basic measurement tool used in the polygon testing of radars is the surveying Epoch 50 GNSS receiver from Spectra Precision. The receiver interoperates with the Nomad type field controller, including the installed Survey Pro field measurement software. The receiver features 220 receiver channels and can operate with the use of the following signals:

- GPS L1/L2/L2C/L5;
- GLONASS L1/L2.

**Figure 21:**
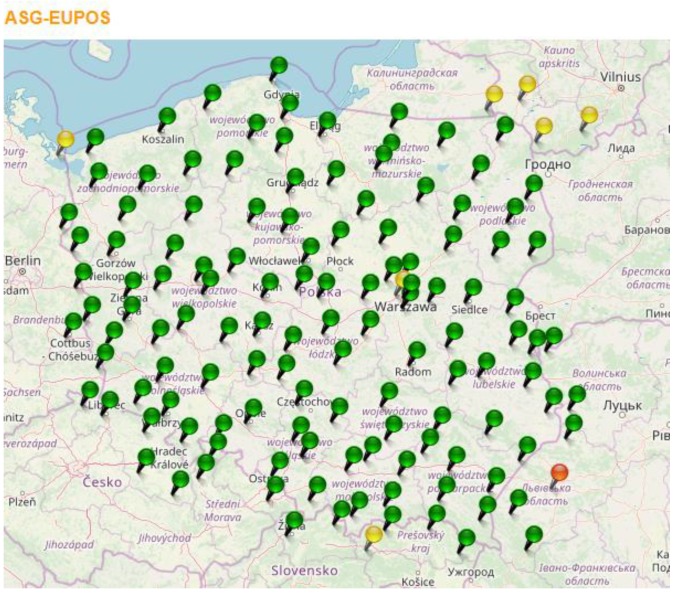
Distribution of the GNSS reference stations of the ASG-EUPOS network. (www.asgeupos.pl [11]).

**Figure 22:**
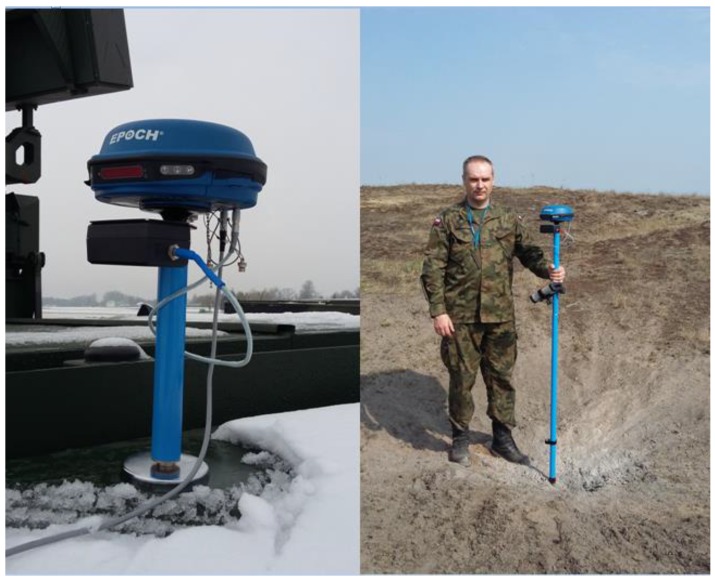
Application of the Epoch 50 GNSS receiver during field measurements.

The Epoch 50 can operate in the following modes: autonomous, code differential, and phase differential. The differential modes can be realized in real time with the use of differential corrections from the local reference station (radio modem) or with the use of corrections sent via the Internet, for example, from the networks of the ASG-EUPOS reference stations. During field testing practice, the most common solution is the ability to record the measurements with a single receiver and their latter specification in the Spectra Precision Survey Office software. Such a specification only requires downloading from the ASG-EUPOS network of the files including the data recorded by the reference station located nearest to the testing location. The scheme of distribution of the reference stations available in the ASG-EUPOS network is presented in Figure 21. The receiver is used to obtain precise coordinates of the field of position of the tested radar, launcher positions, and the points of impact of the ballistic objects (Figure 22) [10].

**Figure 23:**
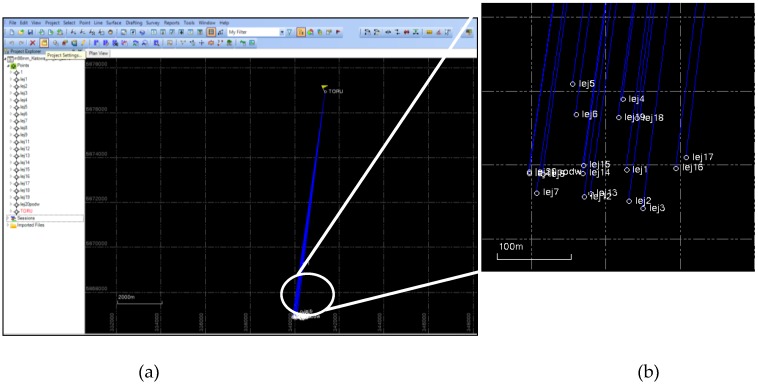
Presentation of the GPS measurements in the Spectra Precision Survey Office software: (**a**) the main program window; (**b**) oom on measuring points.

Figure 23 presents an illustrative box in the Spectra Precision Survey Office software with visible measurement points specified in the post processing mode, in relation to the TORU permanent reference station.

## 3. Results

Research and testing of modern radars are complicated processes, both in technical and organizational terms. One of the dominant trends in radiolocation is the development of multifunctional radars. Such radars can detect and identify the following: aircrafts, helicopters, unmanned flying objects, and small high-speed ballistic objects. They require comprehensive knowledge of various fields of technology and a constant quest for new research methods from the research teams [12]. In particular, the specificity of field testing, conducted with the use of various types of launchers requires, apart from specialist knowledge in the domain of radiolocation, knowledge of artillery operations, the specificity of military training areas, and experience in using satellite geodesy. Our presented methods made it possible to check radar parameters with small high-speed ballistic object detection function accurately and objectively.

Technological progress in the field of radiolocation contributes not only to the improvement of the basic technical parameters of radars, forcing an increase in the accuracy of existing research and testing methods, but also leads to the creation of completely new functionalities [13].

In this presentation of parameters, we are aware that this article leaves unsatisfied the specific properties and test results of individual devices, but this is impossible due to their final military application.
